# Sequencing analysis of the SCA6 CAG expansion excludes an influence of repeat interruptions on disease onset

**DOI:** 10.1136/jnnp-2017-317253

**Published:** 2018-01-24

**Authors:** Sarah Wiethoff, Emer O’Connor, Nourelhoda A Haridy, Suran Nethisinghe, Nicholas Wood, Paola Giunti, Conceição Bettencourt, Henry Houlden

**Affiliations:** 1 Department of Molecular Neuroscience, UCL Institute of Neurology, London, UK; 2 Center for Neurology and Hertie Institute for Clinical Brain Research, Eberhard Karls-University, Tübingen, Germany; 3 Department of Neurology and Psychiatry, Faculty of Medicine, Assiut University Hospital, Assiut, Egypt; 4 Department of Clinical and Experimental Epilepsy, UCL Institute of Neurology, London, UK

**Keywords:** Spinocerebellar ataxia type 6, polyglutamine diseases, repeat interruptions, trinucleotide repeat expansions, disease modifying factors

## Introduction

Polyglutamine (polyQ) diseases are caused by coding expanded (CAG)n repeats in the respective genes.^1^ Repeat instability, both germ line and somatic, is known to occur in most polyQ diseases and can influence the phenotype. Longer repeats are usually associated with earlier ages at onset (AAO) and/or more severe disease courses. Repeat instability with expansion bias can have severe clinical consequences to patients with these devastating conditions for which there are no disease-modifying treatments available. Several factors, including the size of the repeats (longer repeats are more unstable), and the presence of sequence interruptions (pure repeats are more unstable) are known to have an influence.^2^

Spinocerebellar ataxia type 6 (SCA6), a late-onset autosomal dominant relatively slowly progressive and pure cerebellar ataxia,^3^ is one of the six polyQ SCAs. SCA6 is caused by expanded (CAG)n repeats in *CACNA1A*. Normal alleles range from 4 to 18 CAG repeats, while expanded repeats range from 20 to 33. Interestingly, SCA6 expanded alleles lie within the normal range of repeat sizes for all other polyQ diseases, below their respective pathogenic thresholds (usually 35–40 repeats or over).^1^ The *CACNA1A* repeats seem to be more stable than other CAG repeats.^3^ The presence of repeat interruptions can enhance overall repeat stability and has been observed in several polyQ diseases (eg, SCA1 and SCA2).^4^ Variants in DNA repair genes significantly modify AAO in polyQ diseases (eg, *RRM2B* SNPs in SCA6),^1^ and it has been hypothesised that this common genetic mechanism could operate through somatic expansion of repeats. Whether repeat purity/interruption plays a role in this association and possibly acts in synergy with repeat length and DNA repair gene variation remains undetermined for SCA6 and is subject of this report, together with genotype–phenotype correlations and AAO data in a previously undescribed UK SCA6 cohort.

## Subjects and methods

**Figure 1:**
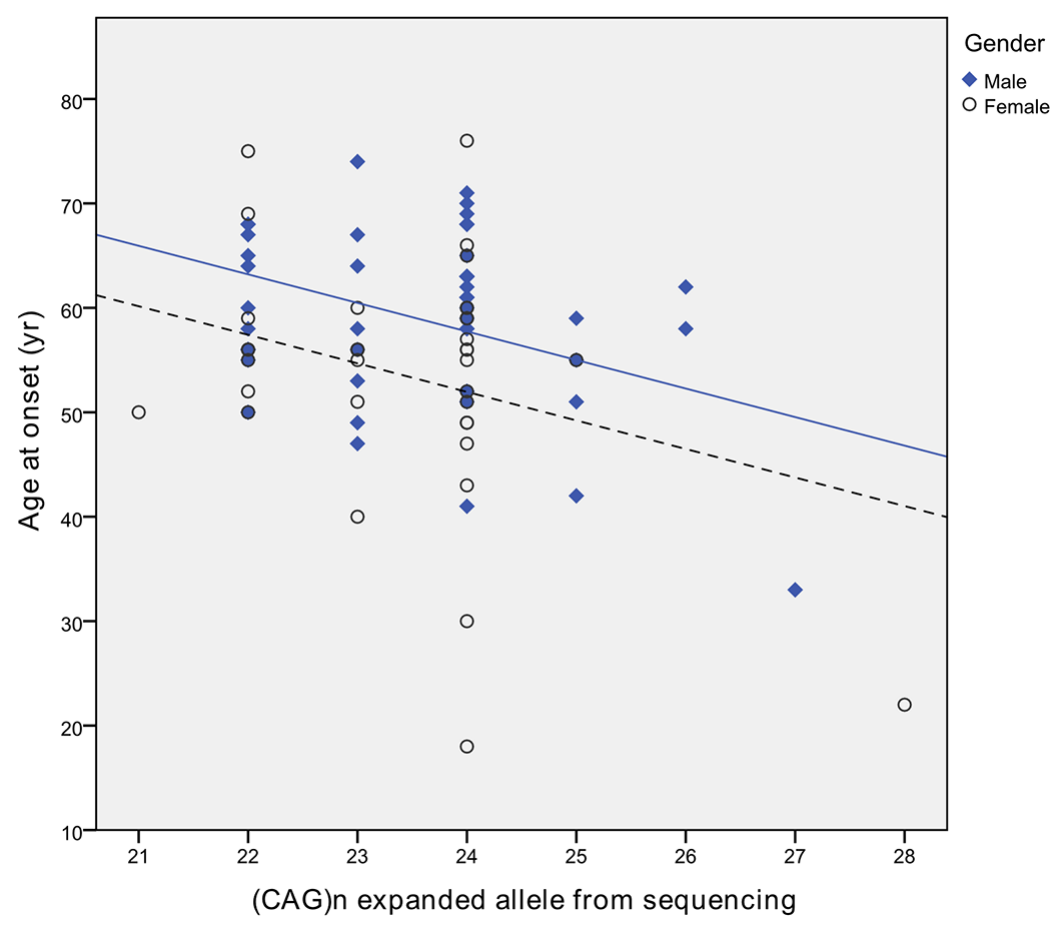
Relationship between spinocerebellar ataxia type 6 (SCA6) age at onset and the size of the expanded alleles. Scatter plot showing the inverse relationship between SCA6 age at onset and the *CACNA1A* (CAG)n repeat size in the expanded alleles. The difference between males and females is highlighted with the corresponding tendency lines being shown (solid line for males and dashed line for females).

A total of 173 DNA samples from a UK SCA6 patient cohort (including all samples from the DNA repair association study^1^) were gathered. The SCA6 (CAG)n repeat was directly Sanger sequenced in all samples (details available in the methods in the online Supplementary file 1). Forward and reverse sequences were thoroughly inspected for the presence of CAG interruptions and repeats were sized (see figure 1 in the online Supplementary file 2). Allele sizes were compared with those obtained previously for diagnosis by fragment analysis. Clinical features were retrospectively analysed for a subset of the SCA6 cohort, with the AAO being retrieved for 86 patients. The relationship between the AAO, the (CAG)n allele size and other predictors was evaluated by correlation and multiple linear regression using SPSS V.22. P values <0.05 were considered significant. The patients gave informed consent.

10.1136/jnnp-2017-317253.supp1 Supplementary file 1

10.1136/jnnp-2017-317253.supp2 Supplementary file 2

## Results

Quality sequences allowed visual inspection for interruptions in the whole SCA6 repeat tract (see figure 1 in the online Supplementary file 2), but no repeat interruptions were observed. The allele size distribution is presented in the figure 2 in the online Supplementary file 3. An average difference of +1 CAG repeat (range −1 to +4) was obtained in the sequencing sizing of expanded alleles when compared with fragment analysis, suggesting that fragment analysis underestimates the repeat size as previously reported for other repeats.^4 5^

10.1136/jnnp-2017-317253.supp3 Supplementary file 3

The average AAO in this SCA6 cohort was around 57 years (see table 1 in the online Supplementary file 4). A significant inverse correlation was found between the AAO and the expanded allele size (figure 1), explaining about 9.1% (F (1,84)=8.423, P=0.005) of the variance in AAO. Gender was also a significant contributor, improving the AAO explanation to 20.3% (F (3,80)=6.792, P<0.001), when accounted for in addition to repeat sizes. AAO was on average 5 years earlier in females than males (53.5±11.9 (SD) vs 58.9±8.3 (SD); t (82)=2.479, P=0.015).

10.1136/jnnp-2017-317253.supp4 Supplementary file 4

## Discussion

The importance of assessing the presence/absence of repeat interruptions arises from their influence on patients’ clinical features (eg, AAO) and also its capability to modulate further expansions in the germ line, with potential implications for genetic counselling. Interruptions of the (CAG)n tract were not observed in our 173 SCA6 patients and are therefore unlikely to play a role in this disease and/or stability of the SCA6 repeats. Similarly to spinal bulbar muscular atrophy,^5^ SCA6 can be distinguished from other polyQ diseases where repeat interruptions have been observed (eg, Menon *et al* 4). Our study excludes the possibility that repeat interruptions could have influenced the results of the previous association study between SCA6 AAO and variants in DNA repair machinery,^1^ as interruptions were ruled out in all patients with SCA6 included in that study. As characteristic for polyQ diseases (eg, Betterncourt *et al* 1), we observed an inverse correlation between the size of the expanded alleles and SCA6 AAO. However, it was considerably weaker than in previous SCA6 studies (figure 1).^6^

These results in a large SCA6 cohort show for the first time that the trinucleotide repeat expansions in *CACNA1A* are pure CAG repeats. It is worth pursuing repeat sequencing in other repeat diseases though, to understand whether they are potentially reducing the statistical power to detect disease-modifying factors, which might constitute valuable potential therapeutic targets.
