# Supplementary material for: Sequencing analysis of the SCA6 CAG expansion excludes an influence of repeat interruptions on disease onset
Source: J Neurol Neurosurg Psychiatry. 2018 Jan 24;89(11):1226–7. doi: 10.1136/jnnp-2017-317253 (PMC6227801; doi:10.1136/jnnp-2017-317253)
Supplement: Supplementary file 1 [file jnnp-2017-317253supp001.pdf]

SUPPLEMENTARY METHODS

A touch-down PCR was performed with 5 µl FastStart PCR Master (Roche), 0,5 µl forward primer (5'-CACGTGTCCTATTCCCCTGTGATCC-3', c=10 µM), 0,5 µl reverse primer (5'-TGGGTACCTCCGAGGGCCGCTGGTG-3', c=10 µM), 2 µl ddH_20, 1 µl DMSO, and 1 µl DNA using the following cycling conditions: 12 mins at 94 °C, followed by 25 cycles of 30 secs at 94 °C, 30 secs at 65 °C (lowered in each cycle by ~0.2°) and 45 secs at 72°C, followed by 13 cycles of 30 secs at 94 °C, 30 secs at 60 °C and 45 secs at 72°C, and a final 10 mins at 72 °C. PCR products were inspected via gel electrophoresis and purified by enzymatic removal (Exosap). Cleaned PCR products were incorporated in a sequencing reaction (ingredients: 2 µl Sequencing Buffer, 1 µl forward or reverse primer, 0.5 µl BigDye®, 3 µl ddH_20, 3,5 µl cleaned PCR product), and sequenced on a 3730 DNA analyser (Applied Biosystems). Obtained chromatograms were analysed using the demo version of Sequencher 5.1 (Gene Codes Corporation, Ann Arbor, MI). Repeat sizes for the normal and expanded allele were determined by thorough analysis and manual counting of visualised repeats on forward and reverse sequencing using BioEdit v7.2.5 (Tom Hall, Ibis Biosciences, Carlsbad, CA 92008) and Sequencher (demo version of Sequencher 5.1, Gene Codes Corporation, Ann Arbor, MI). Normal and expanded allele were read separately and obtained sequence was compared to the *CACNA1A* wildtype sequence in order to investigate the possibility of presence of repeat interruptions. When a discrepancy for the repeat count between forward and reverse existed, the longer repeat was taken consistently.
