# Supplementary material for: Sequencing analysis of the SCA6 CAG expansion excludes an influence of repeat interruptions on disease onset
Source: J Neurol Neurosurg Psychiatry. 2018 Jan 24;89(11):1226–7. doi: 10.1136/jnnp-2017-317253 (PMC6227801; doi:10.1136/jnnp-2017-317253)
Supplement: Supplementary file 2 [file jnnp-2017-317253supp002.pdf]

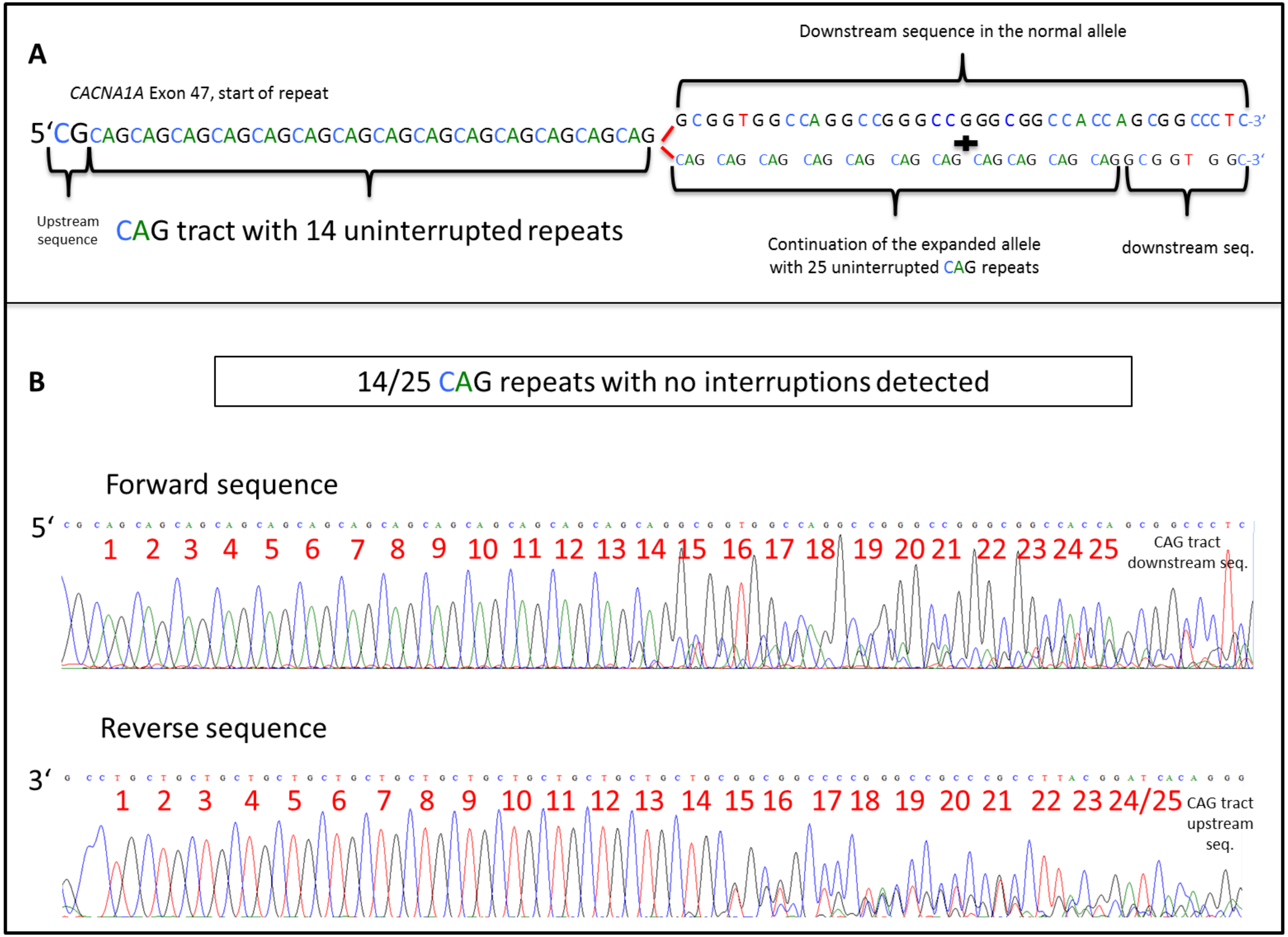

Supplementary Figure 1: Exemplary *CACNA1A* (CAG)n repeat in a SCA6 patient with a 14/25 genotype.
A) Schematic representation of *CACNA1A* normal and expanded (CAG)n alleles and surrounding upstream and downstream sequences (seq.). B) Electropherograms obtained by direct Sanger sequencing of the *CACNA1A* (CAG)n repeat. The forward sequence shows 14 uninterrupted CAG repeats counting from the 5'-end of the repeat tract, while the reverse sequence shows 14 uninterrupted CAG repeats counting from the opposite end (3'-end). By visually inspecting both forward and reverse sequences the presence of interruptions could be excluded in the whole CAG repeat tract. Whenever there was a discrepancy in the size of the expanded alleles between forward and reverse, the longest allele was consistently considered.
