# Supplementary figures and images for: Sequencing analysis of the SCA6 CAG expansion excludes an influence of repeat interruptions on disease onset
Source: J Neurol Neurosurg Psychiatry. 2018 Jan 24;89(11):1226–7. doi: 10.1136/jnnp-2017-317253 (PMC6227801; doi:10.1136/jnnp-2017-317253)

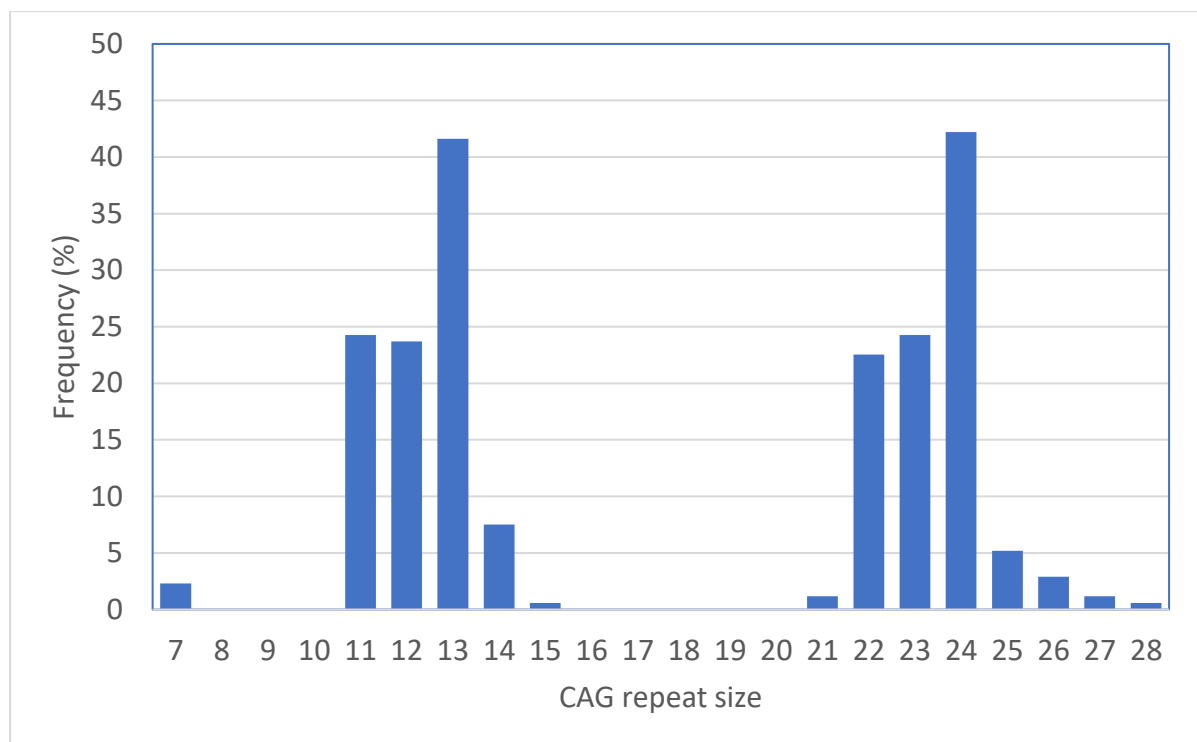

**Supplementary Figure 2: *CACNA1A* CAG repeat size distribution in a SCA6 UK cohort (N=173).**

Supplement: Supplementary file 3 [file jnnp-2017-317253supp003.pdf]
