# Supplementary material for: Sequencing analysis of the SCA6 CAG expansion excludes an influence of repeat interruptions on disease onset
Source: J Neurol Neurosurg Psychiatry. 2018 Jan 24;89(11):1226–7. doi: 10.1136/jnnp-2017-317253 (PMC6227801; doi:10.1136/jnnp-2017-317253)
Supplement: Supplementary file 4 [file jnnp-2017-317253supp004.pdf]

Supplementary Table 1: Characterization of the UK SCA6 cohort

**Features**	**Overall cohort (N=173)***	**AAO subcohort (N=86)**
**Mean normal allele size ± SD [range]**	12.2 ± 1.2 [7-15]	12.4 ± 1.1 [7-15]
**Mean expanded allele size ± SD [range]**	23.4 ± 1.1 [21-28]	23.5 ± 1.2 [21-28]
**Interruptions detected?**	No	No
**Mean AAO (yrs ± SD) [range]**	---	56.7 ± 10.2 [18-76]
**Gender (M/F, %)**	54/46[a]	57/43[b]

***This cohort is mostly composed of unrelated individuals.**
Abbreviations: SD=standard deviation; AAO=age at onset; yrs=years; M=male; F=female; [a]Missing for 5 individuals; [b]Missing for 2 individuals.
